# Surgical Problems in the Aged

**Published:** 1980

**Authors:** 


### Book Reviews

### SURGICAL PROBLEMS IN THE AGED
**K. D. J. Vowles, John Wright, Bristol**
**£9.00**

This is a highly readable book. It may also be the first book to deal specifically with the special problems of surgery in the aged. That nobody has thought of doing this before is most surprising in view of the plethora of books on all aspects of medicine and of the relevance of the subject to nearly every sort of surgeon.

The author has enlisted the support of other colleagues in Exeter and they cover general surgery, orthopaedic surgery, thoracic surgery and urology besides medical assessment, anaesthesia and limb fitting. Perhaps the most important chapter deals with postoperative problems. Inevitably in a work by 6 authors concentrating on the same theme there is a tendency towards repetition but it is the important messages that are hammered home and this is no bad thing. One can detect a variety of literary styles but in only one of these is it anything but lucid and fluent.

For the reviewer the message of the book is that the elderly will tolerate almost any operation provided it is the right operation carried out at a carefully chosen moment and that a prolonged series of half measures are likely to fail. This book is highly recommended.

